# In Silico and In Vitro Analyses of LncRNAs as Potential Regulators in the Transition from the Epithelioid to Sarcomatoid Histotype of Malignant Pleural Mesothelioma (MPM)

**DOI:** 10.3390/ijms19051297

**Published:** 2018-04-26

**Authors:** Anand S. Singh, Richard Heery, Steven G. Gray

**Affiliations:** 1 Thoracic Oncology Research Group, Trinity Translational Medical Institute, St. James’s Hospital, Dublin D08 W9RT, Ireland; rheery@tcd.ie (A.S.S.); anandsimarsingh@gmail.com (R.H.); 2 MSc in Translational Oncology Program, Trinity College Dublin, Dublin 2, Ireland; 3 HOPE Directorate, St. James’s Hospital, Dublin 8, Ireland; 4 Department of Clinical Medicine, Trinity College Dublin, Dublin 8, Ireland; 5 Labmed Directorate, St. James’s Hospital, Dublin 8, Ireland

**Keywords:** malignant pleural mesothelioma, long non-coding RNAs (lncRNAs), epithelial- mesenchymal transition

## Abstract

Malignant pleural mesothelioma (MPM) is a rare malignancy, with extremely poor survival rates. At present, treatment options are limited, with no second line chemotherapy for those who fail first line therapy. Extensive efforts are ongoing in a bid to characterise the underlying molecular mechanisms of mesothelioma. Recent research has determined that between 70–90% of our genome is transcribed. As only 2% of our genome is protein coding, the roles of the remaining proportion of non-coding RNA in biological processes has many applications, including roles in carcinogenesis and epithelial–mesenchymal transition (EMT), a process thought to play important roles in MPM pathogenesis. Non-coding RNAs can be separated loosely into two subtypes, short non-coding RNAs (<200 nucleotides) or long (>200 nucleotides). A significant body of evidence has emerged for the roles of short non-coding RNAs in MPM. Less is known about the roles of long non-coding RNAs (lncRNAs) in this disease setting. LncRNAs have been shown to play diverse roles in EMT, and it has been suggested that EMT may play a role in the aggressiveness of MPM histological subsets. In this report, using both in vitro analyses on mesothelioma patient material and in silico analyses of existing RNA datasets, we posit that various lncRNAs may play important roles in EMT within MPM, and we review the current literature regarding these lncRNAs with respect to both EMT and MPM.

## 1. Introduction 

Malignant pleural mesothelioma (MPM) is a rare, but aggressive form of cancer, predominantly associated with prior exposure to asbestos [1]. Whilst many countries have banned the use of asbestos [2], it is still used in developing countries. A recent report based on extrapolations for asbestos use estimated global mesothelioma deaths at 38,400 per annum [3], and while there have been some recent advances in this disease, particularly with respect to immune-oncology [4,5], the current standard of care (a combination of pemetrexed/raltitrexed and cisplatin chemotherapy) [6,7] is non-curative, and results in a response rate of approximately 40% [8].

Epithelial–mesenchymal transition (EMT) is a process by which epithelial cells shed many of their epithelial traits and acquire various features observed in mesenchymal cells. During this transition, epithelial cells lose their polarity and many of their intercellular contacts, such as desmosomes, adherens junctions, and tight junctions, resulting in their disassociation from epithelial sheets. At the end of this process, cells undergoing EMT assume a variety of mesenchymal-like properties: enhanced migratory capacity, invasiveness, heightened resistance to apoptosis, and greatly increased production of extracellular matrix components [9].

Most MPMs have three main histologic subtypes, divided into epithelioid, sarcomatoid, or mixed (biphasic) [10,11]. However, multiple morphological patterns have also been described within these subtypes, and similarities in clinical presentation and histological appearance of MPM, primary lung carcinoma, pleural metastases, reactive pleural diseases, and rare pleural malignancies can pose challenges to MPM diagnosis [12]. Indeed, “The current gold standard of MPM diagnosis is a combination of two positive and two negative immune-histochemical markers in the epithelioid and biphasic type, but sarcomatous type do not have specific markers, making diagnosis more difficult.” [12]. Because MPM has a partial fibroblastic phenotype in the context of EMT, it has been postulated that this may, in part, explain the aggressiveness of this cancer conferring both high invasiveness and chemoresistance [13], and in this regard, it may be applied to the epithelioid versus sarcomatoid histotype of MPM [13]. In this regard, the epithelioid and sarcomatoid histologic variants of MPM can be considered as E- and M-parts of the EMT axis, with the biphasic histotype considered an intermediate [14]. In support of this, hierarchical clustering of transcriptomic data from MPM separates this cancer into two distinct molecular subgroups, and one subgroup (C2) with an associated EMT molecular signature has worse overall survival (OS) [15].

A significant body of work has examined the roles of other forms of non-coding RNA such as microRNAs in both EMT [16,17] and MPM [18,19], and there is some evidence that miRNAs and lncRNAs interact or cross-talk to orchestrate EMT [20]. Despite the known roles of lncRNAs in the establishment of EMT in cancer, a topic recently reviewed in detail by us and others [21,22], very few studies have specifically examined the functional roles of lncRNAs in MPM [23,24,25,26].

With the advent of high-throughput sequencing technology, transcriptomic data for MPM is emerging. Using unsupervised consensus clustering of RNA-seq-derived expression data from 211 MPM samples, Bueno et al. [27] identified four major clusters: sarcomatoid, epithelioid, biphasic-epithelioid (biphasic-E), and biphasic-sarcomatoid (biphasic-S). Of these, differential expression analysis of the sarcomatoid and epithelioid consensus clusters identified a significant number of lncRNAs which could distinguish between these, as shown in Table 1.

A discussion of the putative roles for these and other lncRNAs in EMT will be presented in subsequent sections.

In this manuscript, we examined the expression of a novel series of lncRNAs (Epidermal Growth Factor Receptor- antisense RNA 1 EGFR-AS1, prostate cancer associated transcript 6 PCAT6 and zinc finger E-box binding homeobox 2 antisense RNA 1 ZEB2-AS1) for altered expression in MPM. We show that all three of these lncRNAs are overexpressed in MPM, and that one of them, PCAT6, is significantly altered across all of the histological subtypes.

Subsequently, using in silico meta-analysis of existing The Cancer Genome Atlas (TCGA) and other datasets (www.cbioportal.org; http://watson.compbio.iupui.edu/chirayu/proggene/database/?url=proggene; www.oncomine.org), we review the known lncRNAs previously described by us and others in MPM (PVT1, NEAT1, PAX8-AS1, and GAS5). Finally, using in silico analyses, combined with a review of the current literature, we examine additional lncRNAs with known roles in EMT for the dysregulated expression in MPM, and show that for many of these, this dysregulated expression is often associated with the biphasic histological subtype. These results suggest that many lncRNAs may be a factor in the transition from the epithelioid to the more aggressive sarcomatoid histotype of malignant pleural mesothelioma.

## 2. Results

### 2.1. Novel LncRNAs with Altered Expression in MPM

Several lncRNAs have recently been identified by our unit as having potentially significant roles in MPM. In the following sections we describe their expression and putative roles in both EMT and MPM.

#### 2.1.1. EGFR-AS1

**Figure 1:**
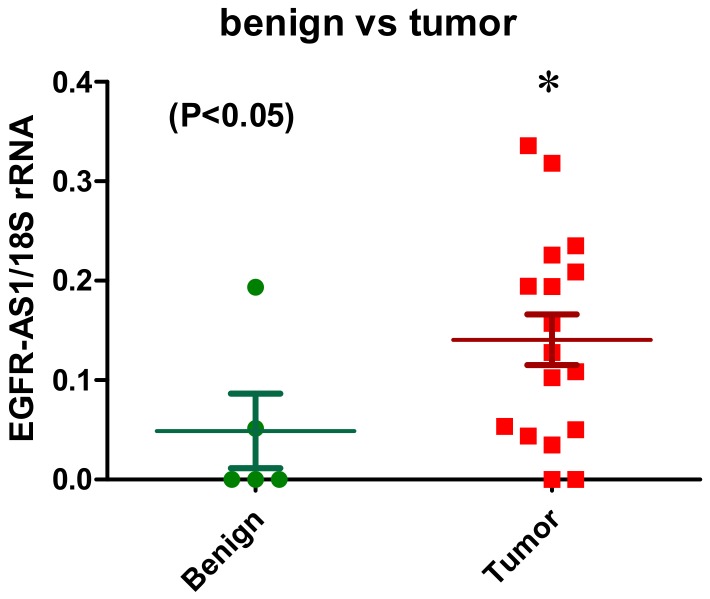
Overexpression of EGFR-AS1 in primary malignant pleural mesothelioma (MPM). EGFR-AS1 lncRNA expression was examined by RT-PCR in a series of primary MPM (*n* = 17) versus benign pleura (*n* = 5). Semi-quantitative densitometric analysis of the results determined that EGFR-AS1 lncRNA was significantly elevated in the tumours compared to benign pleura. Statistical significance was assessed using a 1-tailed unpaired Students *t*-test (* *p* = 0.0445).

High expression of EGFR is associated with MPM [38]. However, clinical trials of EGFR tyrosine kinase inhibitors (TKIs) as single agents in MPM failed [39,40,41]. However, more recently, expression of EGFR on MPM has been used for the targeted delivery of microRNA mimics delivered by targeted bacterial minicells (TargomiRs) in a recent clinical trial in MPM [42], while most recently a patient harbouring mutations in EGFR (G719C and S768I) was successfully treated with Afatinib an EGFR TKI [43]; an lncRNA associated with EGFR called EGFR-AS1 has been identified. This lncRNA was shown to regulate EGFR expression in liver cancer [44], and most recently, expression of this lncRNA has been shown to be associated with sensitivity to EGFR TKIs in patients with head and neck SCC (HNSCC) [45]. Strikingly, knockdown of EGFR-AS1 in vitro and in vivo lead to increased sensitivity, whereas overexpression is sufficient to induce resistance to EGFR TKIs [45]. In this regard, preliminary data from our group has shown that EGFR-AS1 is significantly overexpressed in MPM (Figure 1); this may explain in part why EGFR TKIs failed as single agents in clinical trials of MPM. The role of EGFR-AS1 in EMT has as yet to be determined. However, the known role of EMT in bypassing EGFR dependence [46] suggests that this lncRNA may indeed play a role in orchestrating EMT transitions in MPM.

#### 2.1.2. PCAT6

PCAT6 is a lncRNA linked to KDM5B (also known as JARID1B). This lysine demethylase has been shown to induce EMT in various cancers, including lung cancer [47,48,49]. Expression of PCAT6 has also been shown to be altered in NSCLC [50,51], and circulating levels of this lncRNA in patient blood has potential as both a diagnostic and prognostic biomarker in NSCLC [51].

**Figure 2:**
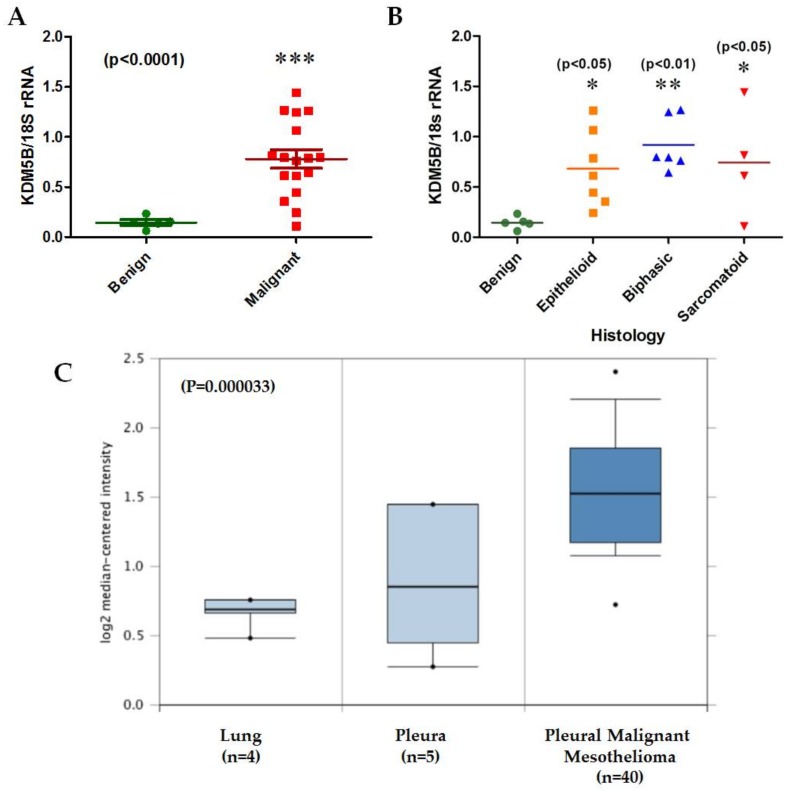

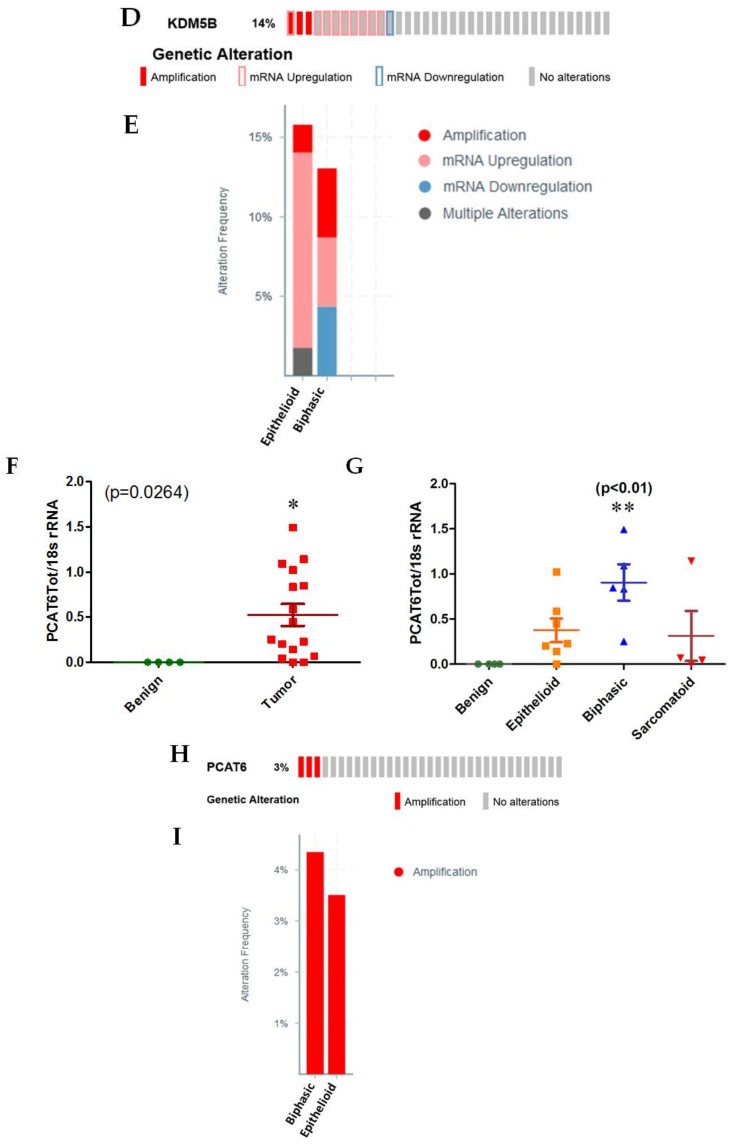
An examination of KDM5B and PCAT6 expression/alterations in MPM. (**A**) KDM5B mRNA is significantly elevated in tumours (*n* = 17) compared to benign pleura (*n* = 5), (**B**) the same samples stratified by histological subtype, (**C**) Oncomine analysis of the Gordon mesothelioma dataset confirming significant overexpression of KDM5B, (**D**) in silico examination using cBioPortal reveals that 14% of samples had alterations to KDM5B, (**E**) when stratified by histotype, these alterations were restricted to epithelioid or biphasic subtypes, (**F**) total PCAT6 lncRNA is significantly elevated in tumours (*n* = 16(red) compared to benign pleura (*n* = 4—green), (**G**) when stratified by histological subtype (Benign = green; Epithelioid = yellow; Biphasic = blue; Sarcomatoid = red), elevated expression of total PCAT6 is significant only in the biphasic subset. Statistical significance was assessed using a Mann–Whitney *t*-test (* *p* < 0.05), or by an ANOVA using Dunnett’s Multiple Comparison Test (* *p* < 0.05; ** *p* < 0.01; *** *p* < 0.001), (**H**) in silico examination using cBioPortal reveals that 3% of samples had amplification of PCAT6, (**I**) when stratified by histotype, these alterations were restricted to biphasic or epithelioid subtypes.

Preliminary data from our group indicates that expression of KDM5B is significantly upregulated in primary MPM (Figure 2A), remaining significant across all histological subtypes (Figure 2B). Similar significant overexpression of KDM5B is also observed in MPM samples in the Gordon et al. [52] mesothelioma dataset (Figure 2C). Across the TCGA dataset, KDM5B appears to have significant alterations in about 14% of MPM cases, including amplification of its genomic region, overexpression, or indeed downregulation of its mRNA (Figure 2D), all of which are found in either the epithelioid or biphasic subtypes (Figure 2E).

Moreover, we have also shown that PCAT6 itself is upregulated in MPM (Figure 2F). However, when examined across histological subtypes, the upregulation observed was significant only in the biphasic subset (Figure 2G). In the TCGA dataset, expression of this lncRNA does not appear to be upregulated, although amplification of its genomic location occurs in 3% of MPM specimens (Figure 2H), again, similar to KDM5B, these are spread over the epithelioid and biphasic subsets. (Figure 2I).The functional role of this lncRNA in EMT is as yet unknown, but knockdown of this lncRNA in lung cancer is associated with inhibited cellular proliferation and metastasis [50].

#### 2.1.3. ZEB2-AS1

ZEB2 is a known regulator of EMT [21]. Originally called ZEB2NAT, but now more often described as ZEB2-AS1, this natural anti-sense lncRNA of ZEB2 was shown to regulate ZEB2 during the process of EMT [53]. This lncRNA has been found to be upregulated in both urinary bladder cancer [54] and hepatocellular carcinoma [55], and in bladder cancer cells is partly responsible for activation of ZEB2 during EMT induction by Transforming growth factor beta (TGF-β) [54]. Furthermore, knockdown of this lncRNA in Hepatocellular Carcinoma (HCC) cells results in reduced vimentin and N-caherin expression with restoration of E-cadherin expression [55], further supporting a role for this lncRNA in the regulation of EMT.

**Figure 3:**
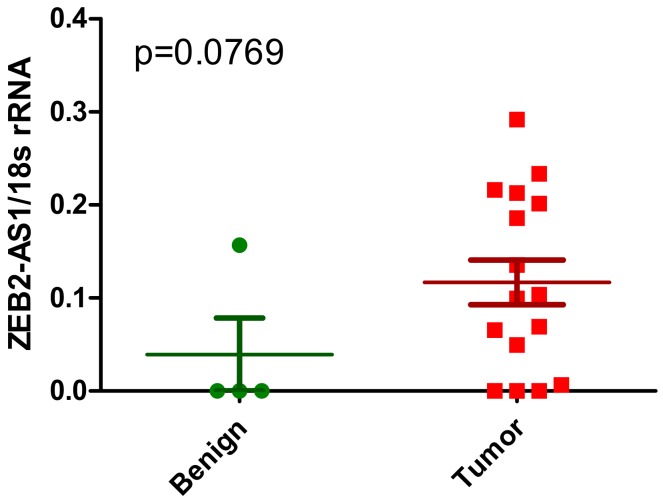
Altered expression of ZEB2-AS1 in primary MPM. ZEB2-AS1 lncRNA expression was examined by RT-PCR in a series of primary MPM (*n* = 16—red) vs. benign pleura (*n* = 4—green). Semi-quantitative densitometric analysis of the results suggests that ZEB2-AS1 lncRNA was elevated in the tumours compared to benign pleura. Statistical significance was assessed using a 1-tailed unpaired Students *t*-test (*p* = 0.0769).

ZEB2 was found to be a significantly altered gene between the sarcomatoid vs. epithelioid clusters (unadjusted *p*-value: *p* < 2.03 × 10^−26^) in the analysis by Bueno et al. [27], but this has not been supported by earlier analysis in the Gordon dataset [52]. There is some suggestion that in the larger dataset by Lopez-Rios that higher expression of ZEB2 is associated with the sarcomatoid subtype (*p* = 0.065) [56]. Very little is known about the expression of ZEB2-AS1 in MPM. Our preliminary analysis suggests that expression of this lncRNA is potentially dysregulated in MPM (Figure 3), but further studies will be required to validate these observations.

### 2.2. Previously Published lncRNAs with Known Links to MPM

A significant body of research has shown that many short non-coding RNAs, such as microRNAs (miRNAs), have extensive alterations and diverse roles in MPM, and have been discussed by us in depth in a previous review [18]. The evidence for altered expression or roles of lncRNAs in MPM has not as yet been exhaustively analysed in MPM. In the following sections we review the current knowledge of the known lncRNAs associated with MPM, and whether or not these lncRNAs can be linked to EMT processes.

#### 2.2.1. PVT1 and c-Myc

PVT1 is an lncRNA which has been shown to be associated with poor prognosis in many cancers [57]. Its expression has also been linked to EMT in various cancers. For example, in breast cancer, PVT1 is significantly upregulated, and directly interacts with SOX2 to drive EMT [58]. In pancreatic cancer, PVT1 has been found to promote EMT by downregulation of the cyclin-dependent kinase p21 [59]. The other ways PVT1 has been shown to elicit responses include by acting as a competitive endogenous RNA (ceRNA) for various miRNAs [60,61,62,63,64,65,66,67], or by interactions with EZH2 to epigenetically regulate genes associated with EMT [68,69,70,71,72,73,74,75].

Both PVT1 and c-Myc are located at the same chromosomal location (8q24.21) and an increase in PVT1 expression is required for high MYC protein levels in 8q24-amplified human cancer cells [76]. In this regard, frequent coamplification and cooperation between c-MYC and PVT1 oncogenes have been observed to promote malignant pleural mesothelioma [24]. Next Generation Sequencing (NGS) demonstrated a downregulation of PVT1 in a sarcomatoid subset compared to epithelioid (Table 1) [27].

**Figure 4:**
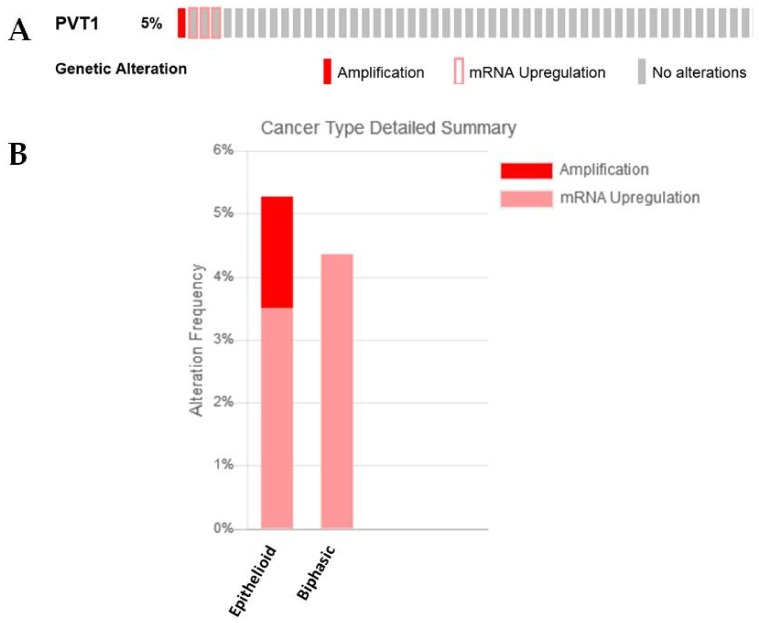
An examination of PVT1 expression/alterations in the TCGA dataset. (**A**) In silico examination using cBioPortal reveals that 5% of samples had overexpression of PVT1 RNA, (**B**) when stratified by histotype, only the epithelioid subtype had amplification of PVT1, whereas some patients with epithelioid and biphasic but not sarcomatoid subtypes had overexpression of this lncRNA.

In silico analysis of the TCGA provisional dataset demonstrated that amplification occurred only in epithelioid samples (Figure 4A,B), which is somewhat in agreement with the observations made by Riquelme et al., where copy number gains were seen in the biphasic (6 of 26, 23%) and epithelioid (5 of 37, 13%) histotypes but not in the sarcomatoid cases [24]. In samples where PVT1 overexpression is observed it is either associated with the epithelioid or biphasic histology (Figure 4B).

#### 2.2.2. NEAT1

**Figure 5:**
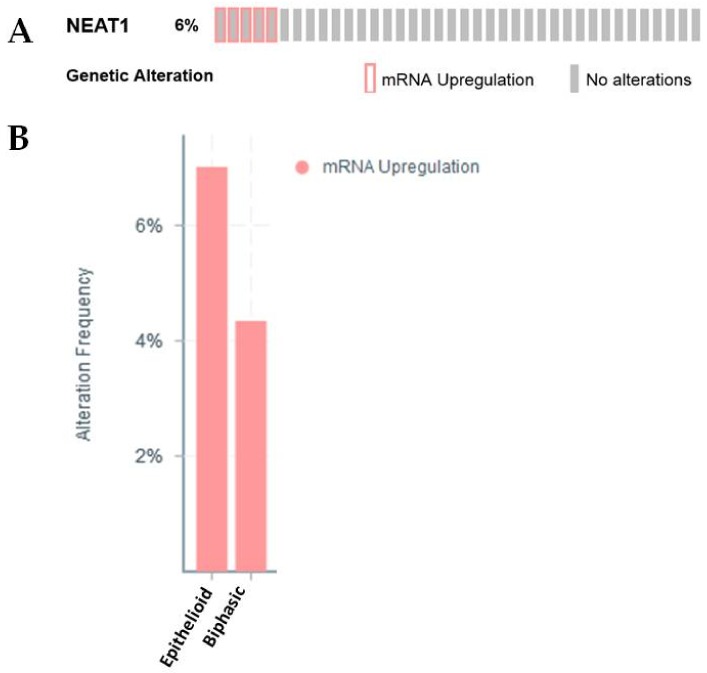
An examination of Neat1 expression/alterations in the TCGA dataset. (**A**) In silico examination using cBioPortal reveals that 6% of samples had overexpression of Neat1 lncRNA. (**B**) When stratified by histotype, the majority of samples with elevated Neat1 are found in the epithelioid subset, followed by a proportion in the biphasic subset.

Neat1 was identified by our group as an lncRNA altered in MPM [26]. It is now well-established that this lncRNA promotes EMT [21,77], and one of the means by which it affects EMT is through regulation of EZH2 [78,79]. Most recently, the expression of NEAT1 has been shown to be BAP1 dependent [80]. Given that it is estimated that approximately 65% of mesotheliomas harbour mutations inactivating BAP1 [81], this may have implications with respect to the role of this lncRNA in MPM pathogenesis. Both our data, and that of Bueno et al. (Table 1) [27], showed an overall downregulation of this lncRNA in MPM. Further analysis of the TCGA dataset shows that a proportion of samples have upregulation of this lncRNA (Figure 5A), which when stratified by histology, is found mostly in the Epithelioid subset, with a smaller proportion in the Biphasic subset also showing elevated expression (Figure 5B).

#### 2.2.3. PAX8-AS1

This lncRNA was also identified [26] as being significantly altered in MPM. The gene associated with this lncRNA, *PAX8*, has been shown to play important roles in the development of ovarian cancer [82], and may do this through upregulation of markers of EMT [83]; although conflicting results have emerged [84]. Interestingly, PAX8 expression is observed in peritoneal mesotheliomas [85,86], but not in pleural mesotheliomas [86]. In MPM, no significant changes in expression of PAX8 were seen in the Gordon dataset [52], whereas high expression of PAX8 was observed in 4 of 87 MPM samples (5%) in the TCGA dataset (data not shown).

Whilst our previous publication found that PAX8-AS1 was significantly altered in MPM [26], analysis of the TCGA dataset using cBioPortal found no alterations in this lncRNAs expression, suggesting that perhaps this lncRNA may not play a direct role in the regulation of EMT and/or the pathogenesis of MPM.

#### 2.2.4. GAS5

A link between GAS5 and EGFR TKI sensitivity has also been identified. Levels of GAS5 were downregulated in the EGFR TKI resistant lung adenocarcinoma cell line A549 compared to sensitive cell lines. Moreover, restoration of GAS5 expression could greatly sensitise these cells to gefitinib treatment in xenograft mouse models [87]. In a separate study relating to prostate cancer, increased expression of GAS5 was associated with decreased Akt signalling [88]. Therefore, it could be suggested that lncRNA mediated regulation of Akt signalling seems to highly important in determining the sensitivity of NSCLC cells to EGFR TKI, such as gefitinib.

In MPM, Felley-Bosco and colleagues have shown that this lncRNA is overexpressed in malignant tumours compared to non-tumoural tissue, (* *p*  <  0.0001 expression, Mann–Whitney test) [25]. While loss of this lncRNA is associated with a shortening of the cell-cycle in MPM cell lines, the role of this lncRNA in regulating EMT in MPM is unknown, however, studies in other cancers, such as osteosarcoma, have shown that expression of this lncRNA decreased in tumours compared to adjacent normal tissue. Furthermore, overexpression of GAS5 suppressed cellular proliferation, migration, and EMT in osteosarcoma cell lines [89].

### 2.3. Previously Published lncRNAs with Known Links to EMT

It is now well established that various lncRNAs play essential roles in the regulation of EMT, a subject we recently reviewed in depth [21]. Despite this, several of these key lncRNAs have not been studied in depth in MPM. In the following sections we discuss the known roles of several of these key lncRNAs, and using in silico analyses to describe the current evidence for their altered expression in mesothelioma histological subtypes.

#### 2.3.1. HOTAIR

HOTAIR is a lncRNA transcribed from the HOXC gene cluster that promotes epigenetic silencing of target genes, including the HOXD gene cluster, through the recruitment of the PRC2 and LSD1/CoREST/REST chromatin remodelling complexes [90,91]. It is well established that HOTAIR is overexpressed in a wide variety of solid malignancies, and moreover, that this overexpression is associated with metastasis and tumour recurrence [21]. Critically, HOTAIR has been linked extensively to the promotion of EMT in solid tumours [21]. In this regard, HOTAIR has been found to regulate EMT through recruitment of PRC2 to the CDH1 promoter [92]. HOTAIR also forms a tripartite complex with Snail and EZH2, facilitating the recruitment of EZH2 to Snail binding sites at the promoters of the epithelial genes E-cadherin, Hepatocyte nuclear factor (HNF), HNF1α, and HNF4α, resulting in their epigenetic silencing [93]. HOTAIR also positively regulates the expression of JMJD3 and Snail to regulate EMT [94]. In addition, this lncRNA plays roles in the silencing of many anti-EMT regulators, such as the miRNAs miR-7, miR-34a, and miR-568 [95,96,97].

**Figure 6:**
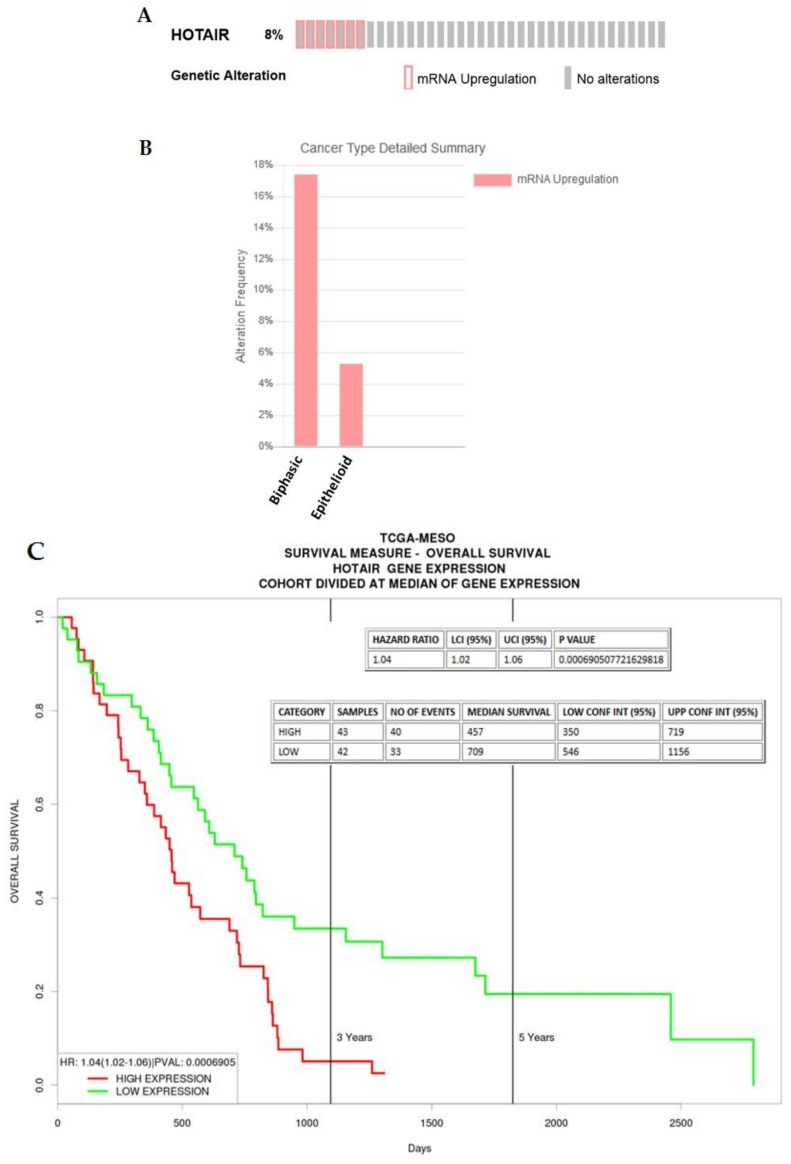
An examination of HOTAIR expression/alterations in the TCGA dataset. (**A**) In silico examination using cBioPortal reveals that 8% of samples had overexpression of HOTAIR RNA, (**B**) when stratified, the majority of these samples were associated with the Biphasic subtype, (**C**) when examined using ProGeneV2 (http://watson.compbio.iupui.edu/chirayu/proggene/database/?url=proggene), higher expression of HOTAIR was associated with a worse overall survival.

In MPM, overexpression of HOTAIR was found in the sarcomatoid subset of the Bueno NGS dataset (Table 1) [27], suggesting that HOTAIR is a lncRNA associated with the progression of MPM from the epithelioid to the sarcomatoid subtype. In silico analysis of an existing TCGA dataset shows that for those samples showing overexpression of this lncRNA the majority were biphasic (Figure 6A,B), and further analysis reveals that higher expression of HOTAIR in mesothelioma is associated with an poorer overall survival (Figure 6C).

#### 2.3.2. MALAT1

MALAT-1 (metastasis-associated lung adenocarcinoma transcript 1 also called NEAT2 or nuclear enriched abundant transcript 2) was first identified in NSCLC as a predictive marker associated with metastatic disease and shorter survival in early stage lung adenocarcinoma [98]. Since its initial discovery, MALAT-1 has been shown to be overexpressed and linked to the promotion of EMT in many cancers [21,99,100]. However, there are conflicting results which suggest that this lncRNA can either promote or inhibit EMT [77,101,102]. This may be in part because MALAT-1 can regulate EMT and other processes in various ways. For example, MALAT-1 can act as a competing endogenous RNA (ceRNA) for various miRNAs including miR-1, miR-200c, miRNA-204, and miR-205 resulting in the subsequent promotion of EMT [103,104,105,106]. Another mechanism by which MALAT-1 induces EMT is via the recruitment of the PRC2 components Suz12 and EZH2 to regulate E-Cadherin [105,107] and β-catenin [108,109].

MALAT-1 has been shown to activate EMT through either MAPK/ERK or PI3K/Akt signalling. MALAT-1 knockdown significantly reduced MAPK/ERK signalling in gallbladder cancer cells [110], and in glioma, MALAT-1 acts as a tumour suppressor by attenuating ERK/MAPK mediated signalling [111]. In osteosarcoma cells, downregulation of MALAT-1 inhibits PI3K/Akt signalling [112], whereas in breast and ovarian cancer cells, knockdown of MALAT-1 knockdown results in increased PI3K/Akt signalling and induction of EMT [102,113]. In this regard, in amodel of silica induced pulmonary fibrosis, MALAT-1 acts as a ceRNA for miR-503, one of whose targets is PI3K p85. By “sponging” this miRNA, MALAT-1 allows stimulation of EMT through a MALAT-1-miR-503-PI3K/Akt/mTOR/Snail pathway [114].

**Figure 7:**
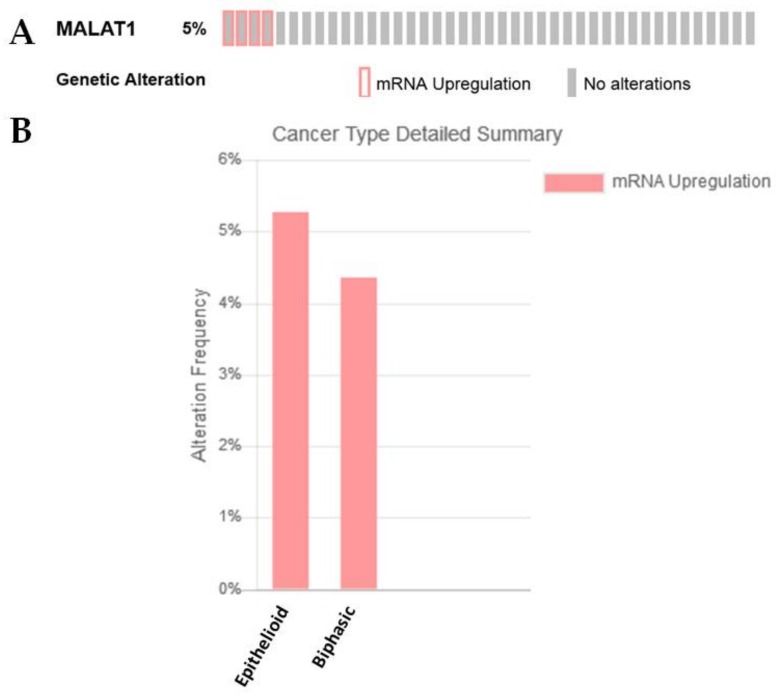
An examination of MALAT1 expression/alterations in the TCGA dataset. (**A**) In silico examination using cBioPortal reveals that 5% of samples had overexpression of MALAT1 RNA, (**B**) when stratified by histotype, the majority of these samples were associated with the epithelioid subtype.

MALAT-1 is induced by TGF-β and plays a critical role during the promotion of EMT by TGF-β in bladder cancer cells [107]. TGF-β often elicits its effect through the Wnt signalling pathway, and significant evidence now suggests that lncRNAs play a major role in this process [115]. For example, MALAT-1 induces EMT in various cancers via the Wnt/β-catenin signalling pathway [116,117,118], while loss of WIF1 enhances the migratory potential of glioblastoma cells through WNT5A activation mediated by MALAT1 [119]. Intriguingly, MALAT1 expression was found to be overexpressed in the sarcomatoid subset of the Bueno NGS dataset (Table 1) [27]. In silico analysis of an existing TCGA dataset also shows that for MPM samples with overexpression of this lncRNA, the majority were epithelioid with some in the biphasic category (Figure 7A,B).

In renal cell carcinoma, a link between MALAT-1 and c-MYC, a downstream effector of Wnt/β-Catenin signalling, was found to be an element in the regulation of β-catenin and transcription factor c-Myc [116]; other lncRNAs have now been shown to play additional roles in regulating EMT via either c-Myc or n-Myc.

#### 2.3.3. MYCNOS and N-MYC

N-Myc (MYCN) belongs to the MYC family and was originally identified as being amplified in 20–30% of neuroblastoma tumours, but it is now well established that dysregulation of this transcription factor is common in many non-neuronal tumours [120]. N-Myc has also been shown to play roles in driving EMT in cancer [121]. In this regard, an lncRNA called MYCNOS has been shown to regulate the expression of N-Myc [122,123,124].

**Figure 8:**
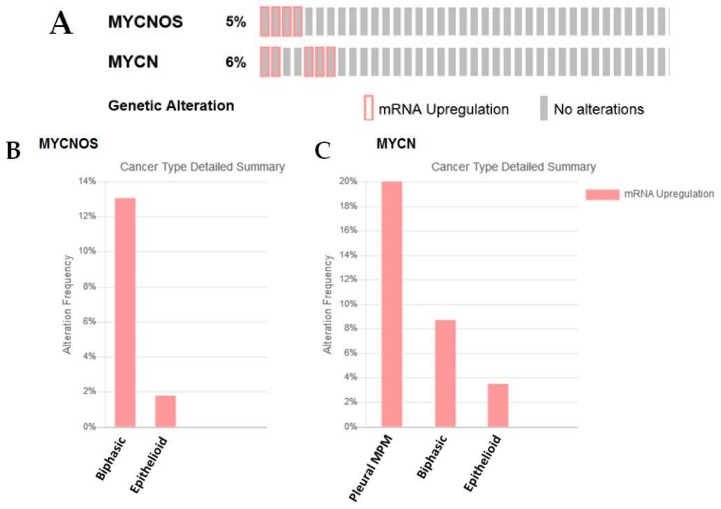
An examination of MYCNOS and N-Myc expression/alterations in the TCGA dataset. (**A**) In silico examination using cBioPortal reveals that 5% of samples had overexpression of MYCNOS RNA, while 6% had overexpression of N-Myc, (**B**) when stratified by histotype, the majority of samples with elevated MYCNOS were found in the biphasic subset, (**C**) N-Myc stratification does not fall into any defined histotype.

While a role for this lncRNA has not yet been identified in MPM, MYCNOS is upregulated in a proportion of MPM (5%—Figure 8A), and is mostly upregulated in the biphasic subset—Figure 8B. N-Myc also shows overexpression in a subset of MPM samples, but the majority of the samples do not fall into a defined histotype (Figure 8C). In these samples only two patients have co-overexpression of both MYCNOS and MYCN.

#### 2.3.4. H19

H19 is an imprinted lncRNA, and has long been identified as an aberrantly expressed non-coding RNA in a great number of cancers, and has been shown to play multi-faceted roles during the tumourigenic process [125]; and is considered to be a critical element in EMT [126]. Indeed, overexpression of this lncRNA is associated with the activation of EMT in numerous cancers, including pancreatic cancer, CRC, nasopharyngeal carcinoma, bladder cancer, gallbladder cancer, and oesophageal cancer4 [21], where it has been shown to silence E-cadherin through recruitment of EZH2 to its promoter, or functions as a ceRNA for several pro-EMT miRNAs [21].

**Figure 9:**
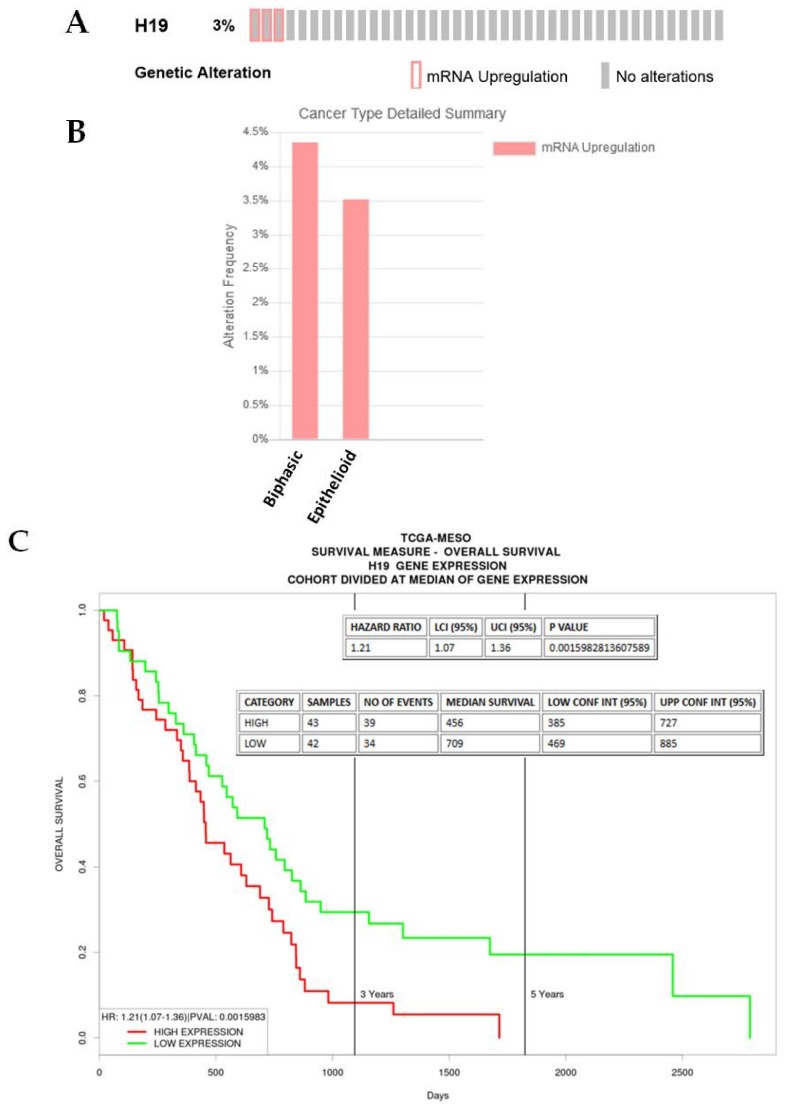
An examination of H19 in MPM. (**A**) H19 lncRNA is altered/overexpressed in a small proportion of MPM patients, as assessed using cBioPortal, (**B**) when separated by histology these samples fall into either the biphasic or epithelioid subsets, (**C**) when overall survival is assessed in this dataset, high median expression is associated with a significantly worse OS (*p* = 0.0016).

Upregulation of this lncRNA is found in the differential analysis between the epithelioid versus sarcomatoid clusters in the analysis by Bueno et al. (Table 1) [27]. In silico analysis of the TCGA dataset suggests that a small number of samples have higher expression of H19 (Figure 9A), which are distributed between the epithelioid (*n* = 1) and biphasic (*n* = 2) (Figure 9B). However, higher median expression of H19 is associated with a worse overall survival in this dataset (Figure 9C).

#### 2.3.5. HULC

The lncRNA Highly Upregulated in Liver Cancer (HULC) was originally identified as one of the most upregulated genes in hepatocellular carcinoma (HCC) [127]; this lncRNA has now been shown to be aberrantly upregulated in several cancers [128]. Some evidence has also been reported suggesting that HULC can also act to inhibit c-Myc expression and PI3K/Akt signalling [129,130], and HULC has also been shown to cooperate with MALAT1 to promote liver cancer stem cell growth/aggressiveness [131]. Moreover, HULC has been shown to affect transcription through interaction with EZH2 [132].

A role for HULC in the regulation of EMT has been observed in HCC where it functions as a ceRNA for miRNAs (miR-122, miR-200a-3p, miR-372, and miR-488) [29,30,133,134] to mediate EMT via upregulation of Snail [135], ZEB1 [29], or ADAM9 [30], and this lncRNA has also been reported to induce EMT in gastric cancer [28].

**Figure 10:**
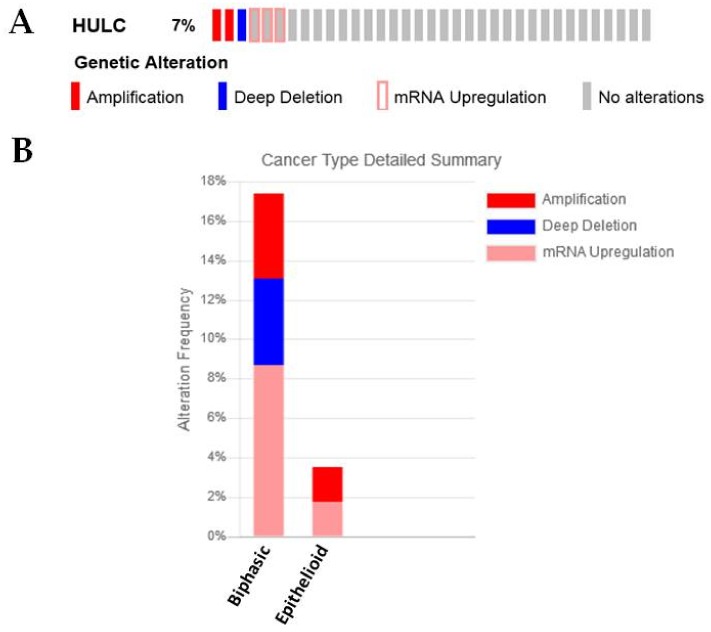
An examination of HULC in MPM. (**A**) HULC is either amplified/deleted or overexpressed in a small proportion (7%) of MPM patients, as assessed using cBioPortal, (**B**) when separated by histology the majority of these samples fall into the biphasic subgroup.

A functional role for this lncRNA in MPM has not yet been identified. However, it was observed to be significantly downregulated in the sarcomatoid compared to the epithelioid subgroup (Table 1) [27]. cBioPortal analysis of the current TCGA mesothelioma dataset finds that 7% of samples have either amplifications or deletions in HULC, or overexpress this lncRNA (Figure 10A). When separated according to histology, the majority of alterations observed were found to be of the biphasic subtype (Figure 10B).

#### 2.3.6. CASC2

In a study of NSCLC, expression of this lncRNA in the adenocarcinoma subtype was associated with inhibition of EMT through regulation of SOX4 [37]. A similar role for this lncRNA in regulating EMT in HCC has been identified, where this lncRNA has been shown to act as a ceRNA for miR-367 via a CASC2/miR-367/FBXW7 axis [136]. Furthermore, CASC2 has been shown to inhibit HCC by acting as a ceRNA for miR-362-5p, which resulted in the inhibition of the Nuclear Factor Kappa Beta (NF-κB) pathway [137].

**Figure 11:**
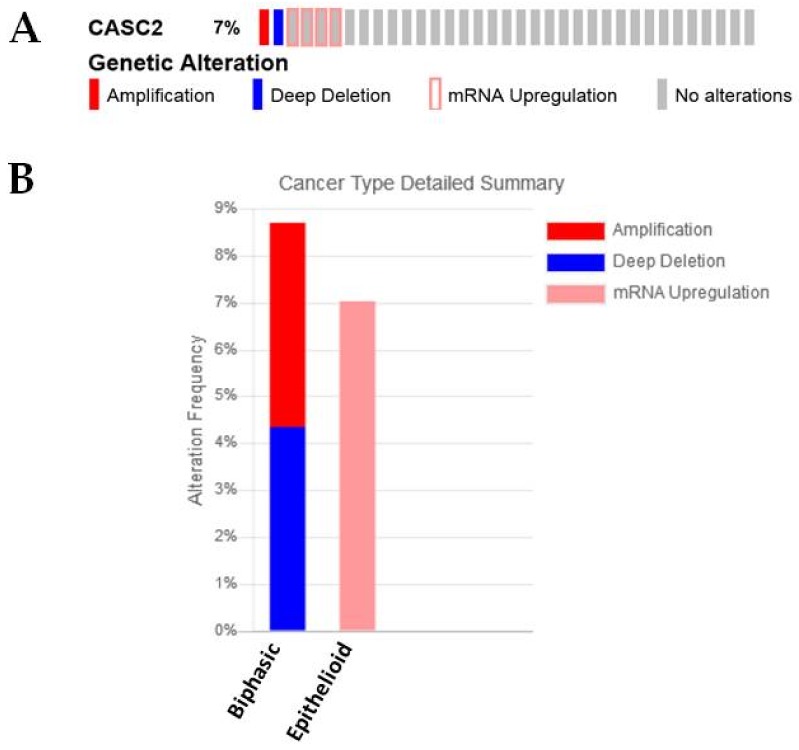

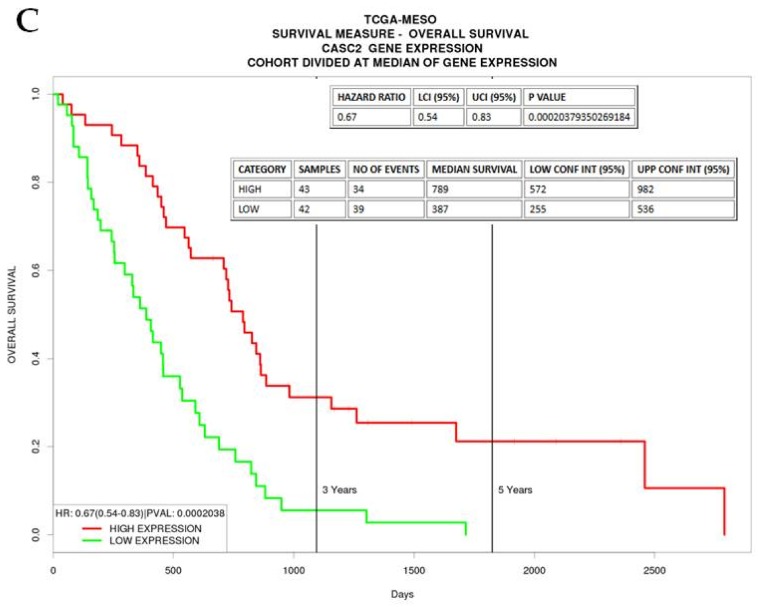
An examination of CASC alterations and expression in MPM. (**A**) CASC2 mRNA is altered/overexpressed in a small proportion of MPM patients as assessed using cBioPortal, (**B**) when separated by histology these samples fall into either the biphasic or epithelioid subsets, (**C**) when overall survival is assessed in this dataset, high median expression is associated with a significantly better OS (*p* = 0.000203).

A functional role for this lncRNA in MPM has not yet been defined, but decreased expression of this lncRNA is significantly associated with the sarcomatoid subtype in the Bueno NGS samples [27] (Table 1). Moreover, analysis of the TCGA dataset in cBioPortal reveals that those samples showing high expression of this lncRNA are associated with more with epithelial and biphasic subtypes, with the majority of the overexpression being observed in the epithelioid subset, while amplifications/deletions of this lncRNA were observed in biphasic samples (Figure 11A,B). When expression of this lncRNA was examined for Overall Survival (OS) benefit using ProGeneV2, high median expression was associated with better overall survival (Figure 11C).

As CASC2 is downregulated in human HCC samples, it may therefore be of interest to examine the levels of this lncRNA in MPM to see if loss of CASC is associated with a more aggressive histological phenotype as observed in Table 1.

#### 2.3.7. ZFAS1

ZFAS1 is a lncRNA transcribed antisense to the ZNFX1 protein-coding gene, first identified as an lncRNA involved in mammary development and subsequently found to have altered expression in breast cancer [138]. Since this initial finding, ZFAS1 has been shown to be pro-tumourigenic and promote EMT in a number of other cancers, including colon cancer, gastric carcinoma, and glioma [33,34,35,36,139,140,141,142,143,144,145,146,147,148].

**Figure 12:**
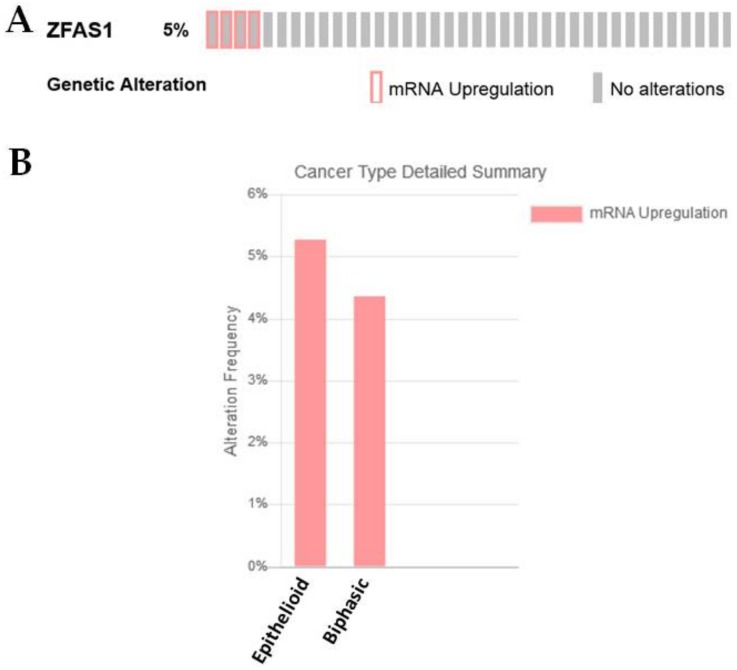
ZFAS1 is altered in a subset of MPM. (**A**) ZFAS1 is overexpressed in a small proportion (5%) of MPM patients as assessed using cBioPortal, (**B**) when separated by histology the majority of these samples fall into epithelioid or biphasic subgroups.

The role of this lncRNA has not yet been identified in MPM, but this lncRNA was found to be significantly altered between epithelioid versus sarcomatoid samples (Table 1) [27]. In the TCGA dataset, ZFAS1 shows overexpression in 5% of the samples; this was associated in samples with epithelioid or biphasic histologies (Figure 12A,B).

## 3. Materials and Methods 

### 3.1. Primary Tumor Samples 

Surgical specimens were obtained as discarded tumour samples from patients who had undergone an extended pleuropneumonectomy at Glenfield Hospital, Leicester, UK. Benign specimens were acquired from patients never diagnosed with MPM. Informed consent was obtained from each patient, and the study was conducted after formal approval from the relevant Hospital Ethics Committee (Leicestershire Research Ethics Committee (REC) references 6742 and 6948). Samples consisted of 5 benign lesions and 17 MPM samples (epithelioid: *n* = 7; sarcomatoid: *n* = 4; biphasic: *n* = 6), details of which are provided in Table 2.

### 3.2. Ethics Statement

Investigations were conducted in accordance with the relevant ethical standards, the Declaration of Helsinki, national, and international guidelines, and were approved by the relevant institutional review board (041018/8804, 13 October 2004, St James’s Hospital/The Adelaide and Meath incorporating the National Childrens Hospital (SJH/AMNCH) REC).

#### Ethics Approval and Consent to Participate

All subjects gave their informed consent for inclusion before they participated in the study. The study was conducted in accordance with the Declaration of Helsinki.

Fresh Frozen Samples: The study was conducted after formal approval from the relevant Hospital Ethics Committee (Leicestershire REC references 6742 and 6948).

### 3.3. RNA Isolation and RT-PCR Amplification

Total RNA was extracted from fresh frozen patient samples using TRI reagent^®^ (Cincinnati, OH, USA) according to manufacturer’s instructions. Prior to first strand cDNA synthesis, 200 ng of total RNA was pre-treated by digestion with amplification grade DNase (Sigma-Aldrich, St. Louis, MO, USA) according to the manufacturer’s instructions. cDNA was then generated using an all-in-one cDNA Synthesis Supermix (Bimake, Houston, TX, USA) according to the manufacturer’s instructions. Patient samples were examined for the expression of various lncRNAs and 18S rRNA at the end point of PCR, using primers and annealing temperatures as outlined in Table 3. Each analysis was carried out once.

PCR cycling conditions were 1 min at 95 °C, 1 min at the appropriate annealing temperature as per Table 2, 1 min at 72 °C for 35 cycles, with a final extension of 72 °C for 10 min. RT-PCR products for each experimental gene and appropriate housekeeping genes (18S rRNA) were run on 2% agarose gels. Following image capture, product quantification was performed using TINA 2.09c (Raytest, Isotopenmeßgeräte GmbH, Straubenhardt, Germany) densitometry software. The mRNA expression was normalised to loading controls, and was expressed as a ratio of target mRNA expression: loading control expression.

### 3.4. Statistical Analysis 

All data are expressed as mean ± SEM unless stated otherwise. Statistical analysis was performed with Prism 5.01 (GraphPad, La Jolla, CA, USA) using either *t*-tests or one-way analysis of variance (ANOVA) where groups in the experiment were three or more. Following ANOVA, post-test analyses utilised the Dunnett’s Multiple comparison test.

### 3.5. In Silico Analysis

In silico analysis was conducted on three additional mesothelioma datasets as follows:

(a) The dataset previously published by Gordon et al. [52], which was interrogated using Oncomine, (b) and an existing TCGA data set (TCGA Mesothelioma; raw data at the NCI; the dataset consists of *n* = 87 samples: epithelioid (57), biphasic (23), sarcomatoid (2), other mesothelioma (5).

Data-mining of the available mesothelioma datasets was conducted using Oncomine [150,151] cBioportal [152,153,154], or PROGgeneV2 [155,156], using their respective default settings.

## 4. Conclusions

Despite intensive efforts, the range of treatment options available to clinicians for the treatment of patients with MPM remains low. The current mainstay of treatment is a combination of cisplatin and pemetrexed (or alternatively raltitrexed), and only approximately 40% of patients will respond to this regimen. At present, no second-line strategy has been approved to date, except rechallenging the patients with long-lasting tumour control after first-line treatment with pemetrexed-based chemotherapy [157].

Across the histological subtypes of MPM, patients who have an epithelioid histology generally have the best OS. Because MPM has a partial fibroblastic phenotype in the context of EMT, it has been postulated that this may, in part, explain the aggressiveness of this cancer by conferring both its high invasiveness and chemoresistance [13]; in particular, with regard to the epithelioid rather than sarcomatoid histotype of MPM [13]. In this regard, the epithelioid and sarcomatoid histologic variants of MPM can be considered as E- and M-parts of the EMT axis, with the biphasic histotype considered an intermediate [14].

In this report, we have shown that many lncRNAs associated with EMT have predominantly altered expression, associated for the most part with the sarcomatoid histologies. Therefore, a greater understanding of the molecular mechanisms governing EMT remains imperative for the development of novel therapies that can slow or prevent metastasis, the current great unmet need of cancer therapy.

In a previous review, we discussed the role of many lncRNAs as elements associated with resistance mechanisms to cisplatin [21], and many of the lncRNAs discussed in this article such as HOTAIR or MALAT1 have well defined roles in cisplatin resistance [21].

If these lncRNAs are both associated with driving MPM from the epithelioid subtype to the more aggressive forms with poorer OS (biphasic and sarcomatoid) with resistance to cisplatin, then potentially targeting these may have therapeutic applicability. Methodologies to restore ncRNAs in MPM, such as the recently completed Phase I MesomiR 1 clinical trial [42], suggest that this technology could also be utilised or adapted to specifically target lncRNAs in MPM.

In conclusion, a large body of evidence suggests that lncRNAs associated with EMT are dysregulated in MPM, and their alteration may be associated with the more aggressive histological subtypes. More work remains to delineate how we may be able to take advantage of this clinically.

## Figures and Tables

**Table 1:** Differentially expressed long non-coding RNAs (lncRNAs) between sarcomatoid versus epithelioid samples as identified by Bueno et al. [27], and discussed in this article.

Name	log2 Fold Change	Unadjusted *p*-Value	Comments
PCAT1	−1.227580845	0.000168412	
HOTAIR	4.342211972	1.09 × 10^−10^	Associates with chromatin remodelling complexes to regulate EMT [21]
MALAT1	−0.902533139	2.72 × 10^−7^	
NEAT1	−0.534058107	0.012990525	Identified as an lncRNA with altered (−2.8 fold) expression in MPM [26]
GAS5	0.053707959	0.785538121	GAS5 shown to have altered expression in MPM
HULC	−0.724711448	0.03946186	Known roles in EMT in other cancers [28,29,30]
H19	2.155715056	1.09 × 10^−9^	Promotes EMT in NSCLC [31], and various other cancers [21]
ZFAS1	−0.443662478	0.018761094	Known regulator of EMT in other cancer settings [32,33,34,35,36]
PVT1	−0.64835701	7.75 × 10^−^^5^	Previously identified as an lncRNA with altered expression in MPM [24]
CASC2	−1.434979397	5.64 × 10^−12^	Overexpression shown to inhibit EMT in lung adenocarcinoma [37]. Associated with Epithelioid and Biphasic samples and high expression associated with better OS in The Cancer Genome Atlas (TCGA) dataset

**Table 2:** Details of pleura/mesothelioma samples used in this study.

Sample	Pathology (Benign, Epithelial, Biphasic, Sarcomatoid)	Age	Gender
JE29	Benign—pleural plaque	55	Male
JE30	Benign—pleural plaque	55	Male
JE32	Benign—pneumothorax	30	Male
JE41	Benign—empyema	68	Male
JE48	Benign—pleural plaque	55	Male
JE31	Epithelioid	62	Male
JE139	Epithelioid	73	Male
JE149	Epithelioid	66	Male
JE155	Epithelioid	56	Female
JE157	Epithelioid	52	Male
JE162	Epithelioid	56	Male
JE173	Epithelioid	54	Male
JE86	Biphasic	54	Male
JE89	Biphasic	54	Female
JE136	Biphasic	41	Male
JE150	Biphasic	58	Male
JE151	Biphasic	N/A	Male
JE160	Biphasic	60	Female
JE106	Sarcomatoid	74	Male
JE125	Sarcomatoid	64	Male
JE133	Sarcomatoid	59	Male
JE145	Sarcomatoid (desmoplastic)	64	Male

N/A—not available.

**Table 3:** Primers and associated annealing temperatures.

Gene/lncRNA	Primer Sequence	Temp	Source
EGFR-AS1	F: 5′-CTTTGCGATCTGCACACACC-3′	62	This study
	R: 5′-GAAGCCTACGTGATGGCCAG-3′		
PCAT6	F: 5′-CCCTAGATACACCCGCCTGGT-3′	64	This study
	R: 5′-ACATTCCAGGGCACCGAGAG-3′		
ZEB2-AS1	F: 5′-GAGAGAGACGAGAGACCCTGAA-3′	60	This Study
	R: 5′-AAATTCATCATGCACACACCC-3′		
KDM5B (JARID1B)	F: 5′-GCTACCCCCTCCAGCTACTCAGA-3′	62	This study
	R: 5′-TCCTCCTCGACTTCCTCCTCATC-3′		
18S rRNA	F: 5′-GATGGGCGGCGGAAAATAG-3′	60	[149]
	R: 5′-GCGTGGATTCTGCATAATGGT-3′		

## Abbreviations

ceRNA	competitive endogenous RNA
EMT	Epithelial Mesenchymal Transition
lncRNA	long non-coding RNA
NSCLC	Non-Small Cell Lung Cancer
miRNA	microRNA
MPM	Malignant Pleural Mesothelioma
NGS	Next Generation Sequencing
OS	Overall Survival
TCGA	The Cancer Genome Atlas
